# Acetic Acid Bacteria: Metabolic Potential, Technological Applications and Emerging Probiotic Functions

**DOI:** 10.3390/foods15132334

**Published:** 2026-07-01

**Authors:** Weronika Głodo, Katarzyna Śliżewska

**Affiliations:** Institute of Fermentation Technology and Microbiology, Faculty of Biotechnology and Food Sciences, Lodz University of Technology, Wolczanska 171/173, 90-924 Lodz, Poland

**Keywords:** acetic acid bacteria, industrial application, bioproduction, oxidative fermentation

## Abstract

Acetic acid bacteria (AAB, family *Acetobacteraceae*) are obligate aerobic microorganisms characterized by a highly efficient oxidative metabolism driven by membrane-bound dehydrogenases. Their ability to incompletely oxidize ethanol and various carbohydrates underlies the production of key food-related metabolites, including acetic acid, gluconic acids, and bacterial cellulose. This review summarizes current knowledge on AAB physiology, metabolic pathways, and ecological adaptations, with emphasis on their relevance to food biotechnology and value-added bioprocesses. AAB plays a central role in traditional and modern food fermentations, contributing to the production of vinegar, cocoa, coffee, kombucha, and other fermented beverages. Their metabolites influence food preservation, sensory attributes, and texture formation, supporting the development of clean-label and sustainable food products. In addition, AAB are increasingly applied in environmental biotechnology, including biodegradation and wastewater treatment, owing to their tolerance to acidic and oxidative stress conditions. Recent advances in metabolic and genetic engineering have enhanced the efficiency, robustness, and product specificity of industrial AAB strains, enabling improved production of organic acids, bacterial cellulose, and other high-value compounds. Emerging evidence also highlights the potential probiotic and postbiotic functions of selected AAB strains, including modulation of gut microbiota, production of bioactive metabolites, and support of intestinal barrier integrity, although these properties remain less explored than in lactic acid bacteria. Despite significant progress, challenges persist in strain standardization, genetic accessibility, and process optimization. Future research should focus on developing advanced engineering tools, improving large-scale fermentation strategies, and further elucidating the functional and health-related properties of AAB. Overall, AAB represents versatile microbial platforms with expanding applications in food science, biotechnology, and sustainable bioprocessing.

## 1. Introduction

**Figure 1:**
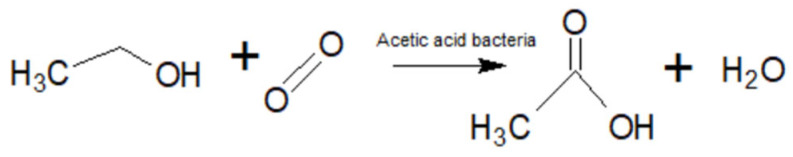
The chemical reaction of the oxidative fermentation of ethyl alcohol to acetic acid. Prepared using program ChemSketch version 12.01.

Acetic acid bacteria (AAB) constitute a specific group of microorganisms of significant biological and economic importance. They are aerobic, Gram-negative bacteria with the unique ability to oxidatively ferment ethyl alcohol to acetic acid (Figure 1). This process forms a basis for vinegar production. It is one of the oldest fermentation products. It has been known and used by humans for thousands of years. Despite their long history of use, AAB remains the subject of intensive scientific research. This research allows for a better understanding of their biology and the development of new biotechnological applications [1,2].

Acetic acid bacteria are common in natural environments rich in sugars and alcohols, such as fruits, flower nectars, juices, fermenting products, and plant surfaces. Their activity contributes to natural fermentation processes and influences the microbiological dynamics of these environments. In addition, the production of acetic acid acts as a natural preservative and contributes to the characteristic flavour and aroma of fermented products [1,3]. In addition to their ecological roles, AAB have become increasingly important in modern biotechnology due to their ability to produce organic acids, bacterial cellulose, and other industrially relevant metabolites [4].

Recent studies have shown that the industrial relevance of AAB extends beyond acetic acid production. They also produce other metabolites of industrial and ecological importance, such as bacterial cellulose, organic acids, and enzymes. The importance of these microorganisms is constantly growing with the development of biotechnology, which increasingly utilizes their properties to produce high-value-added substances and for environmental protection applications [5,6]. AAB represents an interesting model of microorganisms capable of surviving and functioning under conditions of high acid concentrations and other toxic compounds. Adaptive mechanisms enable them to grow and metabolize effectively in such environments. They are the subject of intensive research, which in turn opens prospects for their practical use in processes with increased environmental requirements [1,7].

Understanding acetic acid bacteria (their general characteristics and role in various ecosystems) provides a starting point for further exploration of their taxonomy, morphology, physiology, and industrial and biotechnological applications. This review aims to summarize current knowledge on AAB taxonomy, metabolism, technological applications, and emerging probiotic potential, with particular emphasis on their relevance to food biotechnology and value-added bioprocesses.

**Figure 2:**
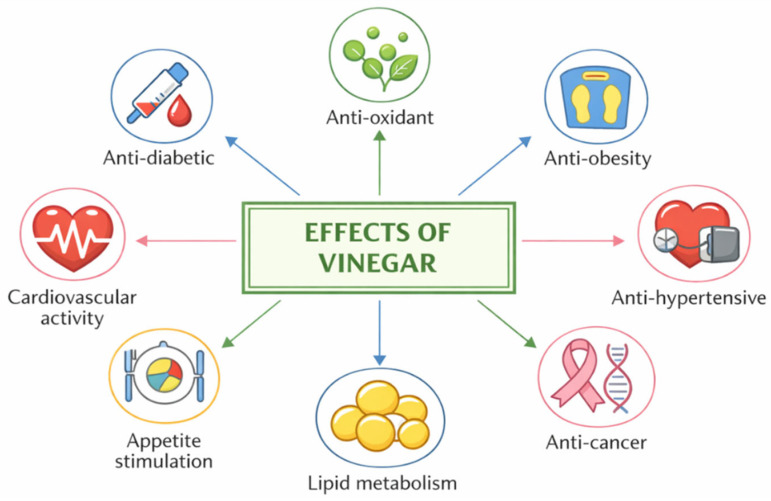
Functional properties and health benefits of vinegar on human metabolism.

The production of acetic acid by AAB has been known since antiquity. Natural alcohol fermentation was used to preserve food and prepare vinegar. It was not until the 19th century (with the development of microbiology) that the role of these microorganisms in fermentation processes was discovered [8,9,10,11,12,13,14,15]. For years, these bacteria were primarily used in the food industry to produce vinegar. It is used both as a condiment and as a preservative [4,16]. The functional properties of vinegar, the main product of AAB fermentation, are important for human health, as shown in Figure 2.

Modern biotechnology is expanding the range of applications of acetic acid bacteria. Beyond their ecological role, acetic acid bacteria have gained considerable importance in modern biotechnology due to their unique oxidative metabolism and ability to produce a wide range of valuable compounds. They are used in the pharmaceutical, cosmetic, environmental, and bioremediation sectors. Of particular interest is the production of bacterial cellulose, a biopolymer with unique physicochemical properties and numerous applications in medicine and industry [5,6,17,18]. Furthermore, AAB are exploited for the production of organic acids and other industrially relevant metabolites [4].

In the natural environment, AAB plays a significant role in organic matter cycling, degrading organic compounds and participating in biogeochemical processes. Thanks to their ability to produce acetic acid, acetic acid bacteria effectively compete with other microorganisms, stabilizing the fermentative microflora. Acetic acid is a strong inhibitor of many microorganisms [7,19,20].

The development of molecular methods and advanced biotechnological techniques is enabling an increasingly detailed understanding of the biology of AAB and their application potential. Research efforts focus on identifying new species, analysing mechanisms of resistance to environmental stress, and optimizing culture conditions and metabolite production [7]. Furthermore, genetic engineering of AAB opens up opportunities to modify their metabolic properties, which can significantly increase the efficiency of acetic acid production and other valuable compounds. However, technological and regulatory challenges remain a barrier that scientists and engineers are striving to overcome to fully utilize the potential of these microorganisms [5,21].

Acetic acid bacteria constitute an important group of microorganisms with broad importance in nature and industry. Their role in natural fermentation processes, unique metabolism, and wide range of biotechnological applications make them an attractive subject for scientific research and practical applications. Understanding their general characteristics and importance is crucial for the subsequent chapters of this article, which will discuss in detail the taxonomy, morphology, metabolism, and applications of AAB [4,22].

## 2. Characteristics, Taxonomy, Morphology, Ultrastructure and Growth Conditions of Acetic Acid Bacteria (AAB)

Acetic acid bacteria, belonging to the class Alphaproteobacteria, constitute an ecologically and taxonomically diverse group of microorganisms capable of oxidizing ethanol to acetic acid through oxidative fermentation. These bacteria were first described in the context of vinegar production, but their biological and industrial significance extends far beyond this function [1,8,21].

**Figure 3:**
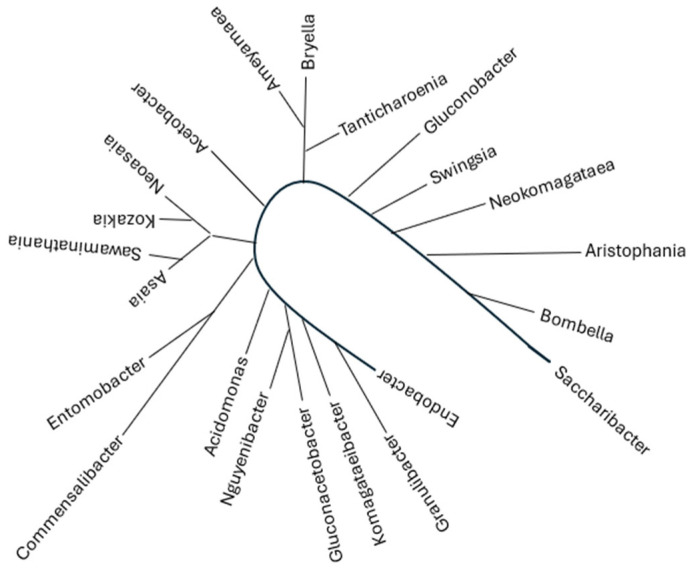
Phylogenetic tree of the AAB genus based on the 16S rRNA gene.

The most important genera of AAB include *Acetobacter*, *Gluconobacter*, *Komagataeibacter*, *Gluconacetobacter*, and *Asaia*, as well as newer and less common taxa such as *Kozakia*, *Saccharibacter*, and *Neoasaia* (Figure 3). Currently, acetic acid bacteria are distributed across multiple genera within the family *Acetobacteraceae*, and their taxonomy is continuously revised due to advances in molecular and genomic methods [6,23,24,25]. Unlike anaerobic fermentation bacteria, AAB are strictly aerobic microorganisms whose metabolism relies on a highly developed system of membrane-bound dehydrogenases involved in the oxidation of alcohols and monosaccharides to the corresponding acids [3,6,16].

Morphologically, acetic acid bacteria are rod-shaped or oval, typically measuring 0.4–1.0 μm wide and 1.0–3.0 μm long. Most are motile due to the presence of flagella [8,26,27]. Unlike anaerobic fermentation bacteria, AAB require oxygen, and their metabolism relies on a highly developed system of membrane dehydrogenases that participate in the oxidation of alcohols and monosaccharides to the corresponding acids [3,6,16].

Acetic acid bacteria exhibit high tolerance to harsh environmental conditions, including high concentrations of organic acids and ethanol, and some strains are capable of surviving at temperatures reaching up to 40 °C. Their ability to adapt to environmental conditions results from a combination of physiological, biochemical, and genetic mechanisms that enable survival under stress. These include the expression of special membrane proteins and antioxidant mechanisms, which allow them to colonize such diverse niches as fruits, plants, flowers, fermenting beverages, and the surfaces of fermentation tanks [1,5,7,28].

*Gluconobacter* differs from *Acetobacter* mainly in its incomplete tricarboxylic acid cycle and limited ability to further oxidize acetic acid to CO_2_ and H_2_O. This metabolic feature makes *Gluconobacter* particularly relevant in fermentations where excessive acidification is undesirable [21,26,29].

Modern AAB taxonomy is based on a polyphasic approach, combining phenotypic and chemotaxonomic data, rRNA gene sequencing, and whole genome sequencing. These methods have allowed for the revision of previous classifications based solely on physiological characteristics, leading to the identification of new genera and a better understanding of the evolution of this group of bacteria [7,23].

### 2.1. Taxonomy of Acetic Acid Bacteria (AAB)

Acetic acid bacteria are a diverse group of aerobic microorganisms classified within the class *Alphaproteobacteria*, order *Rhodospirillales*, and family *Acetobacteraceae*. Over the decades, their classification has been dynamically revised as identification techniques have advanced—from phenotypic characteristics, through chemotaxonomy, to molecular and genetic data [21,23].

Initially, these bacteria were grouped into several broadly defined genera, primarily:

- *Acetobacter*—capable of oxidizing ethanol to acetic acid, and then to carbon dioxide and water (full mineralization);
- *Gluconobacter*—lacking the full Krebs cycle, unable to oxidize acetic acid to CO_2_, which means it remains the primary metabolite [21,26]. However, as genetic and biochemical methods developed, it was discovered that these groups were not sufficiently homogeneous. This led to a systematic revision of the taxonomy, leading to the recognition of additional genera [6,21,23]:

Modern AAB taxonomy is based on the so-called polyphasic approach, including:

- Sequence analyses of rRNA genes (16S, 23S), genes encoding dehydrogenases and membrane enzymes;
- Phenotypic characteristics: the ability to oxidize alcohols, produce acetic acid and cellulose;
- Chemotaxonomy: analysis of membrane fatty acid composition, the presence of ubiquinone Q-10;
- MALDI-TOF typing, DNA–DNA hybridization, and other modern methods.

Some species previously classified in the genus *Gluconacetobacter* have been transferred to the newly created genus *Komagataeibacter*, primarily due to their ability to extensively biosynthesize cellulose and significant genetic differences [6]. Table 1 summarizes the differences characteristic for the genera *Acetobacter*, *Gluconacetobacter*, *Gluconobacter* and *Komagataeibacter*, taking into account morphological, metabolic and taxonomic features.

Similarly, the genera *Asaia*, *Kozakia*, and *Neoasaia* have been separated into separate taxonomic units based on phylogenetic data [30].

Simultaneously, research is being conducted on intrageneric taxonomy—for example, *Acetobacter* exhibits distinct developmental lines, differing in their ability to adapt to fermenting environments and biofilm structure [7,21].

The taxonomy of acetic acid bacteria is not just a matter of systematic classification; it also has real implications for their industrial and biotechnological applications. Knowledge of the properties of specific species and strains allows them to be selected for specific processes: vinegar production, bacterial cellulose production, gluconic acid production, or food fermentation [5,8,29].

### 2.2. Morphology and Ultrastructure of Acetic Acid Bacteria

AAB are characterized by a relatively simple but diverse cellular morphology. They occur primarily as rods (bacilli), less frequently as coccoid-like oval forms, and their length typically ranges from 1 to 3 μm and width from 0.4–1 μm [8,21]. Most representatives of this group are Gram-negative bacteria, possessing the typical cell wall structure of *Proteobacteria*—a thin layer of peptidoglycan and an outer membrane containing lipopolysaccharides (LPS) [8,27].

The typical shape of AAB cells is:

- Rod-shaped or oval, often slightly curved [8,26];
- Occurring singly, in pairs, or less frequently in short chains;
- Some species exhibit pleomorphism—shape variation depending on environmental conditions or the phase of development growth [7,27].

Most AAB species are motile due to the presence of flagella. Their arrangement can be:

- Peritrichous (many flagella surrounding the cell, e.g., *Acetobacter aceti*);
- Polar (1–2 flagella at one end of the cell, e.g., *Gluconobacter oxydans*) [21,26].

This motility is ecologically important, enabling colonization of fermentation surfaces and movement in environments rich in sugars and alcohols [1,7].

**Figure 4:**
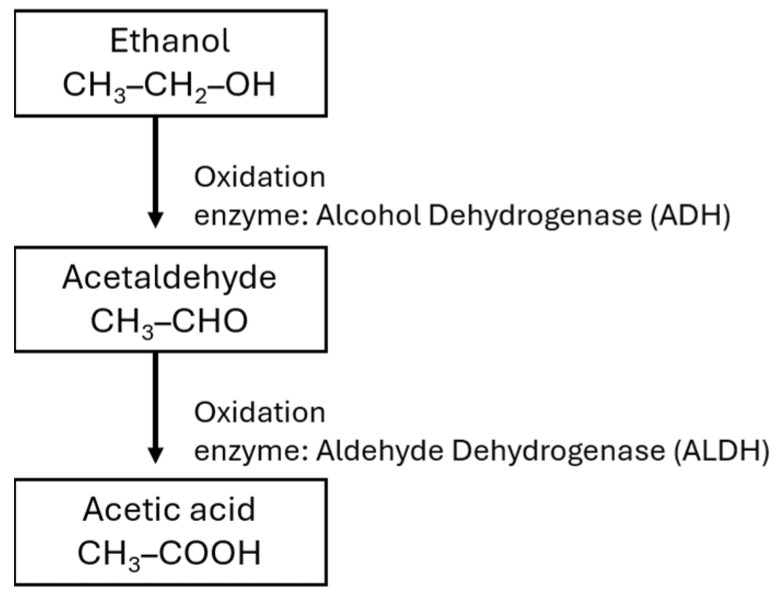
Conversion of ethanol to acetic acid.

One of the most characteristic aspects of the AAB ultrastructure is the intense oxidative activity of the cell membrane, where specific membrane dehydrogenases are located:

- Alcohol dehydrogenase (ADH) and aldehyde dehydrogenase (ALDH) are responsible for the oxidation of ethanol to acetic acid (Figure 4);
- These enzymes are coupled to the electron transport chain, allowing AAB to efficiently utilize energy while simultaneously secreting products into the external environment [31,32].

In *Gluconobacter*, which has limited Krebs cycle activity, metabolism is particularly concentrated in the cell membrane [3,16].

Some species, especially those of the genus *Komagataeibacter*, are capable of synthesizing bacterial cellulose—a highly organized biopolymer that forms a protective matrix around the colony.

- This cellulose serves protective and mechanical functions;
- Plays a role in biofilm formation, including the cellulose-rich pellicle produced by the SCOBY during kombucha fermentation [3,17,18,29,33].

The structure of AAB also includes adaptations that determine resistance to environmental stress, including:

- The presence of heat shock proteins (HSPs) and detoxification systems (glutathione, catalase);
- Increased membrane stiffness at high concentrations of acetic acid and ethanol [29,34];
- Some strains also demonstrate the ability to form spore-like spores, although these are not typical endospores as in Bacillus [26,28].

Using electron microscopy techniques (SEM, TEM) and scanning surface analysis, differences in membrane thickness, the presence of cellular protrusions, and the distribution of enzymatic structures have been identified in different species [6,35]. These differences are important when selecting strains for specific biotechnological applications, for example:

- *Gb. oxydans*—for gluconic acid production;
- *Komagataeibacter xylinus*—for cellulose production;
- *Acetobacter pasteurianus*—for industrial acetic acid fermentation [3,8,36].

The morphology and ultrastructure of acetic acid bacteria reflect their high metabolic specialization and ability to survive in diverse, often extreme conditions. Features such as the presence of specific membrane dehydrogenases, cellulose production, and cell membrane structure are directly responsible for their biological functions and application potential in the food industry, biotechnology, and medicine [6,16,37].

### 2.3. Growth Conditions and Environmental Factors Affecting the Development of Acetic Acid Bacteria

Acetic acid bacteria have specific requirements for environmental conditions that determine their growth, metabolism, and ability to produce acetic acid and other metabolites. Knowledge of these factors is crucial for optimizing fermentation processes in the food and biotechnology industries [1,5,8].

#### 2.3.1. Temperature

AAB are mesophilic microorganisms, with an optimal growth temperature range typically around 25–30 °C [1,8]. Many species can tolerate temperatures up to approximately 37–40 °C, although growth and metabolic activity typically decline above 35 °C. Higher temperatures can lead to denaturation of oxidative enzymes and loss of cell viability [5].

#### 2.3.2. Environmental pH

The optimal pH for AAB growth is in the range of 5.0–6.0 [1,8]. Although these bacteria produce acetic acid, which lowers the pH of the environment, they demonstrate high tolerance to acidic conditions, and the ability to grow at pH values as low as approximately 3.0–3.5 is one of their characteristic adaptive traits. Tolerance mechanisms include the regulation of protective proteins and modifications of cell membrane composition. Under acid stress, AAB alters membrane lipid composition by increasing the proportion of saturated fatty acids and other membrane-stabilizing components, thereby reducing membrane fluidity and permeability to undissociated acetic acid. These adaptations limit intracellular acid accumulation and contribute to the maintenance of intracellular pH homeostasis, enhancing survival under highly acidic conditions [5,28].

#### 2.3.3. Oxygen

AAB are obligately aerobic microorganisms—their oxidative metabolism requires the presence of oxygen as the final electron acceptor [3,8]. Under anaerobic conditions, growth is inhibited or significantly reduced, and acetic acid production ceases. Therefore, acetic acid fermentations are conducted under intensive aeration [28,38].

#### 2.3.4. Carbon and Energy Sources

The main carbon substrate for AAB is ethanol, which the bacteria oxidize to acetic acid and then to carbon dioxide and water [3,8,16]. In addition to ethanol, some species also utilize other alcohols (e.g., glycerol and sorbitol), sugars (glucose, fructose), and organic acids, although with varying degrees of efficiency [16,21].

#### 2.3.5. Humidity and Osmotic Environment

AAB typically inhabit humid environments rich in sugars and alcohols, such as fruits, flower nectars, and fermenting beverages. Although elevated concentrations of sugars and salts may exert osmotic stress and inhibit growth, many AAB species possess adaptive mechanisms that enable survival under fluctuating osmotic conditions commonly encountered during natural and industrial fermentations [1,39,40].

#### 2.3.6. Tolerance to Acetic Acid and Alcohol

One of the key characteristics of AAB is their high resistance to the products of their own metabolism—primarily acetic acid and ethanol [5,28]. Adaptive mechanisms include:

- Changes in the composition of membrane lipids that limit acid permeation;
- Proton pump activity that removes excess H^+^ ions;
- Production of shock proteins (HSPs) that protect enzymes and cellular structures [5].

In addition to these general mechanisms, acetic acid bacteria employ a coordinated physiological response involving modulation of membrane permeability, maintenance of intracellular pH homeostasis, and activation of energy-dependent efflux systems. The increased expression of pyrroloquinoline quinone (PQQ)-dependent dehydrogenases also contributes indirectly to acid and ethanol tolerance by enhancing respiratory activity and energy generation, which is essential for sustaining proton gradients under acidic stress conditions. Furthermore, regulatory networks associated with oxidative stress response are activated under high acetic acid concentrations, allowing cells to maintain metabolic activity and viability in industrial fermentation environments [5,28].

#### 2.3.7. Other Factors Affecting Growth

- Presence of micronutrients and vitamins: AABs require adequate availability of Fe^2+^, Mg^2+^, and B vitamins for the proper functioning of oxidative enzymes [8,21];
- Oxidative Stress: bacteria possess antioxidant systems (catalase, superoxide dismutase) that protect against damage caused by reactive oxygen species [5];
- Microbiological competition: in natural environments, AABs coexist with yeasts, other bacteria, and molds, which influences their growth dynamics [1].

## 3. Acetic Acid and Other Metabolite Production

The production of acetic acid by acetic acid AAB is a biochemical process of crucial importance both for their physiology and for industrial applications such as vinegar fermentation. In this process, ethanol is oxidized to acetic acid by specialized enzymes, including dehydrogenases dependent on cofactors such as PQQ, FAD, and NAD, which are coupled to the bacterial respiratory chain [5,6,41]. The efficiency and specificity of these transformations determine both the production rate and the metabolic profile of the bacteria, including the formation of other important metabolites [16,40].

The efficiency of acetic acid production is strongly dependent on environmental and technological factors, such as the concentration of substrates (ethanol and product), oxygen availability, and medium pH [38,42,43]. AAB exhibits adaptive mechanisms that allow them to tolerate high concentrations of ethanol and ethanol, which is essential for maintaining metabolic activity under stressful conditions [5,28,44]. Furthermore, the level of acetic acid biosynthesis is regulated by complex genetic and metabolic mechanisms [45]. Metabolic engineering strategies, including the overexpression of key oxidative enzymes such as PQQ-dependent alcohol dehydrogenase, together with optimization of fermentation conditions, can improve process efficiency [40,46,47]. A comprehensive understanding of these factors allows for effective control of the production of acetic acid and other metabolites, which directly translates into the development of modern biotechnology technologies [41,44].

**Figure 5:**
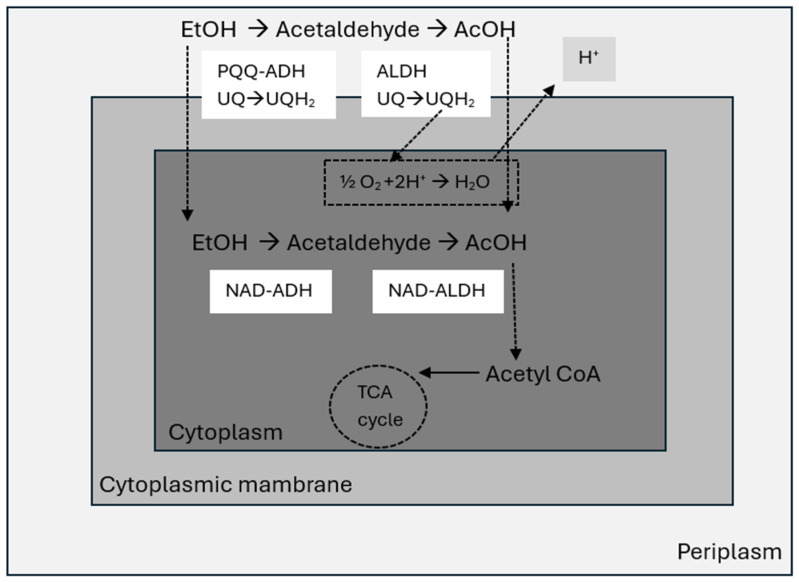
Ethanol oxidation by PQQ-ADH and ALDH at the outer surface of cytoplasmic membrane and by NAD-ADH and NAD-ALDH in the cytoplasm [16].

Acetic acid bacteria convert ethanol to acetic acid through oxidative fermentation, a complex set of enzymatic steps occurring under aerobic conditions. This process not only allows for the efficient oxidation of ethanol to vinegar but also enables the generation of metabolic energy in the form of ATP. Membrane-bound dehydrogenases play a key role in this process—primarily alcohol dehydrogenase (ADH) and aldehyde dehydrogenase (ALDH), whose activity and cofactors determine the efficiency of the entire pathway (Figure 5) [3,6,48].

Step 1: Ethanol oxidation to acetaldehyde

The first step in the metabolism is the oxidation of ethanol (C_2_H_5_OH) to acetaldehyde (CH_3_CHO) by periplasmic alcohol dehydrogenase (PQQ-ADH). This enzyme is bound to the inner membrane and uses pyrroloquinolinequinone (PQQ) as a redox cofactor. Many forms of ADH in AAB are so-called quinoproteins, characterized by high catalytic activity and the ability to transfer electrons to ubiquinone in the respiratory chain [6,41,49]. The presence of PQQ as a cofactor enables a rapid reaction rate and is a key control point in oxidative fermentation [40,50]. Structurally, PQQ-ADH is composed of a catalytic subunit and cytochrome proteins. In *A. pasteurianus*, this enzyme is located on the periplasmic side, allowing for the direct elimination of oxidation products from the cell, thus limiting their toxic impact [49].

Step 2: Oxidation of acetaldehyde to acetic acid

The second stage involves the conversion of acetaldehyde to acetic acid (CH_3_COOH), catalysed by membrane-bound aldehyde dehydrogenase (ALDH). Like ADH, ALDH is a membrane-bound periplasmic enzyme that participates in electron transfer to the respiratory chain [6,16]. Electrons are transferred via ubiquinone to the terminal cytochrome oxidase, generating a proton gradient and enabling ATP production by ATP synthase [35,38,51].

### 3.1. Integration into the Respiratory Chain and Energy Production

The entire ethanol oxidation process is coupled to cellular respiration. Electrons from PQQ-ADH and ALDH pass to ubiquinone and then, via cytochrome complexes, to molecular oxygen as the final electron acceptor. This flow enables the proton pump and the production of energy in the form of ATP by membrane ATP synthase [6,38,47].

The ability of AAB to survive high concentrations of acetic acid and ethanol requires a strong energy potential—hence the importance of efficient respiratory function and the regeneration of PQQ as a cofactor [40,47].

### 3.2. Auxiliary and Alternative Pathways

In addition to the main ADH → ALDH → acetic acid pathway, acetic acid bacteria can also activate other metabolic pathways. Under conditions of reduced oxygen availability, some aldehyde can be metabolized intracellularly, involving cytoplasmic NAD-dependent dehydrogenases. Furthermore, AAB utilize other pathways for carbon source processing, including: Pentose phosphate, Embden–Meyerhof–Parnas (EMP), and Entner–Doudoroff pathways, which play a role in redox balance and the supply of biosynthetic precursors [16].

### 3.3. The Importance of Metabolic Engineering

Modern biotechnology utilizes knowledge of oxidative enzymes in AAB to optimize vinegar production. For example, co-expression of the ADH and ALDH genes, along with PQQ biosynthesis, results in increased ethanol oxidation efficiency and acetic acid tolerance. Changes in the expression of these enzymes significantly impact metabolite flux and the rate of final product production [40,46].

In summary, the stepwise conversion of ethanol to acetic acid in acetic acid bacteria is a highly efficient, aerobic oxidative pathway, whose key components are the membrane-bound ADH and ALDH dehydrogenases and the associated respiratory chain. The efficiency of this pathway depends on the structure of the enzymes, the presence of cofactors (primarily PQQ), and the ability to adapt to environmental stress. Understanding these mechanisms opens up opportunities for metabolic engineering and further optimization of acetic acid bioproduction processes [52].

### 3.4. Production Efficiency—The Impact of Factors Such as Substrate Concentration, Oxygen Availability, and pH

The efficiency of acetic acid production by acetic acid bacteria depends on numerous environmental and technological factors. Although biochemically well understood, this process is highly sensitive to parameters such as ethanol and oxygen concentration, environmental pH, temperature, and the presence of byproducts—primarily acetic acid itself, which can be toxic to bacterial cells [5,38,42,43].

#### 3.4.1. Ethanol Concentration and Its Impact on Fermentation

Ethanol is the main substrate in the oxidative fermentation pathway, and its appropriate concentration is crucial for production efficiency. Optimal ethanol content ranges typically between 4–6%, with higher concentrations (>7%) potentially inhibiting the growth and enzymatic activity of AAB. Ethanol stress induces changes in the structure of cell membranes, leading to impaired permeability and redox potential, which translates into reduced substrate oxidation efficiency [5,42,44].

#### 3.4.2. Oxygen Availability—A Key Condition for Aerobic Fermentation

The oxidative fermentation process is strictly dependent on oxygen availability, which serves as the final electron acceptor in the respiratory chain. Oxygen limitation reduces the activity of membrane-bound PQQ-dependent dehydrogenases (including alcohol dehydrogenase and aldehyde dehydrogenase), thereby decreasing the rate of ethanol oxidation to acetic acid [6,38,43]. Acetic acid bacteria therefore require strongly aerobic conditions, and efficient production is achieved only under high oxygen transfer rates and sufficient dissolved oxygen levels.

Both surface (traditional) and submerged (modern) fermentation systems require controlled aeration; however, oxygen transfer limitations are a key bottleneck in industrial processes. Submerged fermentation systems employ intensive agitation and aeration strategies to improve oxygen supply, resulting in higher productivity compared to surface methods [41,43]. The importance of oxygen availability is closely related to the energy metabolism of AAB and the function of PQQ-dependent dehydrogenases, which rely on continuous electron flow to oxygen as the terminal acceptor [6,38,41,43].

#### 3.4.3. Influence of pH and Acetic Acid

Fermentation efficiency decreases significantly with increasing concentration of the final product—acetic acid. Most *Acetobacter* strains tolerate acidity up to approximately 8–9% *v*/*v* CH_3_COOH, but its presence in the environment leads to a decrease in pH, destabilization of cell membranes, and enzyme inactivation [5,19,20,38,42]. AAB possess adaptive mechanisms, including the AatA pump (efflux transporter), protein chaperones, and lipid modifications of membranes, which allow them to partially counteract the toxic effects of vinegar [5,28,44].

##### Temperature and Other Environmental Parameters

The optimal fermentation temperature for most AAB strains is in the range of 28–32 °C. Too low a temperature inhibits enzyme activity, while too high a temperature can cause denaturation and increase oxidative and acidic stress [5,43]. Furthermore, dynamic temperature changes can disrupt osmotic balance, adversely affecting membrane structure and respiratory activity.

#### 3.4.4. The Role of Cellular Energy (ATP) and Redox Metabolism

The efficiency of metabolic processes also depends on the efficient functioning of the cellular energy system. High ADH and ALDH activity, coupled with ATP production in the respiratory chain, is essential for maintaining the proton gradient, the synthesis of stress proteins, and the functioning of efflux pumps [6,38]. Studies on *A. pasteurianus* strains indicate that increased PQQ synthesis and cellular respiration correlate with higher tolerance to fermentative stress and increased productivity [40,47,52].

##### The Importance of Genetic Modification and Strain Engineering

Fermentation efficiency can also be enhanced through metabolic engineering. Examples include coexpression of genes encoding ADH/ALDH with PQQ biosynthesis, which results in improved ethanol oxidation rates and improved cofactor regeneration [40,46]. Such modifications lead to increased enzymatic activity, reduced accumulation of toxic intermediate metabolites, and more efficient substrate utilization.

The efficiency of acetic acid production by AAB is the result of a complex interaction between environmental parameters (pH, oxygen, temperature), substrate and product concentrations, as well as intracellular regulation of redox metabolism and enzyme activity. Efficient fermentation requires the optimization of these factors and—in a modern approach—the use of strain engineering to maximize productivity and cell resistance to stress conditions.

Acetic acid production by acetic acid bacteria, such as *Acetobacter* and *Komagataeibacter*, is primarily determined by both genetic traits of the production strains and the composition of the culture medium. In recent years, significant progress has been achieved through metabolic and genetic engineering approaches, which have improved both production efficiency and cellular tolerance to ethanol and acetic acid stress [38,40,41,46].

At the molecular level, the efficiency of acetic acid biosynthesis depends mainly on the activity and expression of enzymes involved in ethanol oxidation, particularly periplasmic pyrroloquinoline quinone-dependent alcohol dehydrogenases (PQQ-ADH) and aldehyde dehydrogenases (ALDH), which catalyze the conversion of ethanol to acetic acid [6,16,48]. These enzymes are associated with the inner membrane and function in conjunction with cofactors such as PQQ, FAD, and NAD, enabling electron transfer to the respiratory chain [6,16,41].

Recent studies have demonstrated that overexpression of genes encoding PQQ-ADH, together with enhanced biosynthesis of PQQ, can significantly increase acetic acid productivity and improve stress tolerance in *A. pasteurianus* [40,46,47]. Co-expression of genes involved in ATP regeneration and respiratory chain efficiency further enhances cellular adaptation to fermentation conditions [38,52].

Transcriptomic analyses (RNA-Seq) have identified multiple genes involved in stress response under high acetic acid concentrations, including efflux pumps (e.g., *aatA*), molecular chaperones (Hsp20), antioxidant enzymes (catalase, peroxidase), and membrane-associated proteins involved in maintaining proton motive force [5,44,52].

Metabolic engineering strategies also target accessory metabolic pathways, such as the pentose phosphate pathway and the Entner–Doudoroff pathway, to optimize carbon flux distribution and increase NAD(P)H availability, thereby supporting cellular energy metabolism and biosynthetic capacity [16].

Additionally, the composition of the culture medium plays a key role in process efficiency. The presence of appropriate nitrogen sources (e.g., yeast extract), vitamins (B_1_, B_2_, niacin), and mineral ions (Mg^2+^, Mn^2+^, Fe^2+^) supports enzymatic activity, growth, and overall metabolic performance of AAB, thereby enhancing acetic acid production efficiency [16,41,43].

#### 3.4.5. Genetic Stability and Strain Selection

The genetic stability of production strains is also crucial for long-term process efficiency. Fermentation conducted under stressful conditions (e.g., high temperature, low pH, and presence of inhibitors) can lead to the accumulation of mutations or the loss of plasmids encoding key enzymes. Therefore, strains optimized through adaptive evolution, genetic engineering, or site-directed mutagenesis techniques are used [29,40,47].

## 4. Applications of Acetic Acid Bacteria in the Food Industry

Acetic acid bacteria are an extremely versatile group of microorganisms that play a key role in numerous biotechnological processes in the food industry. Their main characteristic is the ability to oxidize alcohols to acetic acid, which is widely used primarily in vinegar production, but also in the fermentation of many other food products [1,16,53].

Modern technologies for the production of acetic acid and fermented food products utilize both classic and innovative fermentation methods, allowing for the production of high-quality products with a distinctive flavour and aroma [41,54]. AAB are present in natural fermentation processes, such as the fermentation of cocoa, coffee, and other raw materials, where they influence the development of complex sensory profiles and improve the organoleptic properties of the final products [1,55].

Furthermore, metabolites produced by AAB exhibit strong antibacterial and preservative properties, which are fundamental for improving food safety and extending shelf life [19,20,24,56,57]. Natural compounds produced by these bacteria are becoming increasingly desirable in the food industry as an alternative to synthetic preservatives, aligning with trends in the production of “clean” and functional foods [29,58,59]. Table 2 presents selected species of acetic acid bacteria and their corresponding sources in food products in which they play an important fermentative or technological role.

### 4.1. Vinegar Production

**Figure 6:**
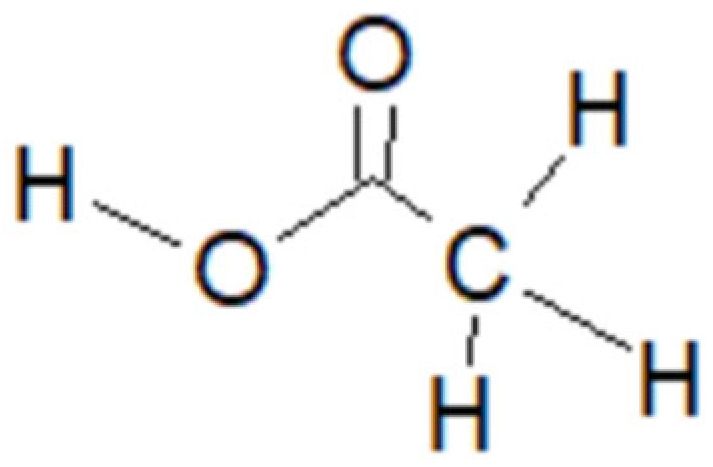
Structural formula of acetic acid. Created using the ChemSketch program.

Vinegar production (Figure 6) is one of the oldest and most widespread uses of acetic acid bacteria in the food industry. This process is based on the biological oxidation of ethanol to acetic acid by specific *Acetobacter* and *Gluconobacter* strains [41,54]. Traditionally, acetic acid fermentation takes place under aerobic conditions, which enables efficient oxidation of ethanol to acetic acid using enzymes such as alcohol dehydrogenase (ADH) and aldehyde dehydrogenase (ALDH) [16,28].

Classical vinegar production primarily involves surface and submerged fermentation. Surface fermentation (e.g., the Orleans method) involves the slow oxidation of alcohol on the liquid surface, resulting in a long fermentation time and lower yield, but providing the vinegar’s characteristic flavour. This method is primarily used to produce higher-quality vinegars, such as wine vinegar or apple cider vinegar [61].

Submerged fermentation, widely used in industry, significantly accelerates the process through intensive oxygen supply and mixing. These systems enable the production of spirit vinegar with high yields and stable parameters. This method is more economically and technologically efficient, making it preferred in large production plants [29,41,54].

Quantitatively, surface fermentation processes typically require significantly longer production times, ranging from several weeks to even 1–2 months, and achieve lower acetic acid productivities, often below 2–3 g/L/h. In contrast, submerged fermentation systems enable much higher process intensification, with fermentation times reduced to a few days and acetic acid productivities reaching approximately 5–15 g/L/h depending on the strain and reactor configuration. These differences are primarily attributed to improved oxygen transfer rates and better control of process parameters in submerged systems, which directly enhance the metabolic activity of acetic acid bacteria [28,41,54].

Technological advances in vinegar production focus on optimizing fermentation conditions, controlling environmental parameters (pH, temperature, substrate concentration), and metabolic engineering of AAB strains [28,41,53]. Modern fermenters are equipped with systems for monitoring and regulating oxygen and pH, which allows for increased product efficiency and quality [29,41].

Additionally, genetic engineering enables the overexpression of key enzymes (e.g., PQQ-ADH) and increasing bacterial resistance to high acetic acid concentrations, resulting in faster and more stable production [28,53,57].

Acetic acid bacteria are used in the production of a variety of food vinegars, differing in their starting raw materials and flavour and aroma profiles. The most popular include:

- Spirit vinegar—produced primarily from rectified ethanol, characterized by a high acetic acid content (approximately 6–8%) and a neutral taste. It is most commonly used as a universal preservative and condiment [41,54];
- Wine vinegar—obtained from the fermentation of grapes or wine must, is distinguished by a mild, aromatic flavour profile, dependent on the raw material and bacterial strain. In the acetic acid fermentation process in wine, *Acetobacter* strains adapted to an environment rich in polyphenols and other phenolic compounds play a crucial role [54,61];
- Apple cider vinegar—produced from apples or apple juice, valued for its characteristic aroma and health benefits. The fermentation process is similar to wine vinegar, but requires specific AAB strains and controlled fermentation conditions to preserve the fruit’s aromatic components [1,54].

Both traditional and modern vinegar production methods rely on the unique properties of acetic acid bacteria, which convert ethyl alcohol into acetic acid while preserving the product’s organoleptic characteristics. The development of fermentation technologies and bacterial metabolic engineering are opening up new possibilities for increasing the yield and quality of vinegars for various applications in the food industry [61,62,63,64].

### 4.2. Fermentation of Cocoa, Coffee, and Other Products

Fermentation is a key step in the production of many food raw materials, such as cocoa, coffee, and other plant-based products. Acetic acid bacteria play a crucial role in these processes, influencing not only the fermentation process but also the final sensory and quality properties of the products [1,53].

During cocoa bean fermentation, AAB interacts with yeast and other microorganisms to oxidize ethanol, produced during the earlier stages of alcoholic fermentation, to acetic acid [55]. This acid acts both as a disinfectant and as a decomposer for the bean pulp, which is essential for developing the typical cocoa flavour and aroma [1,55].

Similarly, in coffee fermentation, AAB participates in the transformation of organic compounds, producing acetic acid and other organic acids, which give coffee its characteristic acidity and enrich the aromatic profile [1]. In both cases, the participation of AAB is closely related to fermentation parameters, such as temperature, oxygen availability, and process duration [41,55].

Metabolites produced by acetic acid, including acetic acid, have a significant impact on the sensory quality of fermented products. In addition to its preservative properties, acetic acid contributes to the development of complex flavour notes appreciated by consumers [1,53]. In cocoa fermentation, proper control of the amount and concentration of acetic acid is crucial, as excessively high levels can lead to undesirable acidity and bitterness [55]. In addition, acetic acid bacteria can produce other aromatic compounds, such as aldehydes and esters, which enrich the flavour profile of the fermented beverage. In coffee, fermentation conducted by AAB can improve aroma intensity and influence the final bouquet of the beverage [53].

Modern fermentation methods involving AAB strive to optimize the process to achieve desired sensory characteristics and microbiological safety. Modern technologies allow for precise control of fermentation conditions, such as oxygen availability and temperature, which influence the microbiota composition and metabolite profile [41,53].

Innovations also include the use of starters containing selected AAB strains, which guarantee repeatability and high quality of fermentation, both in the production of cocoa, coffee, and other fermented products [53,55]. This allows for obtaining products with stable and desirable flavour and aroma properties.

Acetic acid bacteria play a key role in the fermentation processes of many food raw materials, influencing their flavour, aroma, and quality. Advances in fermentation technology involving them allow for increasingly better adaptation of production processes to market requirements and consumer expectations [29,54,60].

Acetic acid bacteria have a significant contribution in improving food quality and safety, primarily through the production of a range of metabolites with antibacterial properties. These metabolites, such as acetic acid, organic acids, aldehydes, and other volatile compounds, exhibit strong antimicrobial activity, which translates into a natural extension of shelf life and protection against pathogens and microorganisms that spoil food products [24,56,57].

Acetic acid, the main metabolic product of AAB, is a well-known preservative that lowers environmental pH, inhibiting the growth of pathogenic bacteria and molds. Its effectiveness against many food pathogens has been confirmed in numerous studies [56,65]. In addition to acetic acid, these bacteria produce other organic acids that synergistically enhance antibacterial activity, as well as metabolites with natural antibiotic properties [56,58].

The mechanisms of action of these compounds include disrupting the integrity of microbial cell membranes, acidifying the cytoplasm, and inhibiting enzymes key to pathogen metabolism [56,66]. As a result, AAB and their metabolites provide an effective barrier against the growth of food-spoiling microorganisms, resulting in longer shelf life [29,59].

The use of acetic acid bacteria as natural biopreservatives is becoming increasingly appreciated in the food industry. Products fermented with AAB, such as vinegar, pickles, and fermented beverages, are very popular due to their natural preservation methods and positive impact on consumer health [1,53,58]. The use of AAB metabolites in food preservation allows for the reduction or complete replacement of synthetic preservatives, which is consistent with the trend towards “clean” and organic food production. Furthermore, these bacteria can be used for biocontrol of food microflora, preventing the growth of pathogens and spoilage microorganisms without negatively impacting the product’s organoleptic properties [29,57].

Modern production technologies using AAB focus on optimizing fermentation conditions and selecting strains with high antimicrobial activity. This allows for increased production efficiency of acetic acid and other metabolites, which translates into improved preservative properties and food safety [41,53,54].

## 5. Probiotic Potential of Acetic Acid Bacteria and Their Role in Modulating the Gut Microbiota

The human gut microbiota constitutes a complex ecosystem of microorganisms that plays a crucial role in maintaining host homeostasis. In recent years, there has been a dynamic increase in the number of scientific publications devoted to the gut microflora, and research findings clearly indicate a strong relationship between intestinal condition and overall human health [67]. Disruptions in the composition of the microbiota, referred to as gut dysbiosis, may lead to the development of numerous metabolic, inflammatory, and immunological diseases [68].

Gut bacteria perform a range of important physiological functions. They are responsible for shaping and modulating the immune system, protecting the host against pathogen colonization, and regulating the secretion of gastrointestinal hormones. Moreover, the gut microbiota is involved in the proper functioning of the gut–brain axis, indirectly influencing the nervous system and neurohormonal processes [67,68].

The importance of the gut microbiota is also reflected in its impact on metabolic processes. Microorganisms inhabiting the gastrointestinal tract participate in the metabolism of nutrients, increasing their bioavailability and influencing lipid and carbohydrate metabolism [68,69]. A properly functioning microbiota may therefore contribute to reducing the risk of civilization-related diseases, such as obesity, type 2 diabetes, and cardiovascular diseases [68].

Another important aspect of gut microbiota activity is its protective role. Through competition for nutrients and adhesion sites, commensal bacteria limit the growth of pathogenic microorganisms [67,69]. Additionally, they stimulate host immune mechanisms, contributing to the maintenance of immunological balance and reducing the risk of inflammatory bowel diseases [69].

In light of current knowledge, increasing attention is being paid to the possibility of modulating the composition and activity of the gut microbiota through diet and the use of live microorganisms with documented health-promoting properties, such as probiotics [47,69]. This interest provides the basis for further consideration of the role of both classical probiotics and next-generation probiotics, including acetic acid bacteria, in maintaining human health [47,67,68,69].

### 5.1. Definition and Classification of Probiotics

According to the widely accepted definition, probiotics are live microorganisms which, when administered in adequate amounts, confer a health benefit on the host [47,67,69]. This definition was formulated by experts from the Food and Agriculture Organization of the United Nations (FAO) and the World Health Organization (WHO), and later refined by the International Scientific Association for Probiotics and Prebiotics (ISAPP) [67,68].

The ISAPP consensus emphasizes that the term “probiotic” applies exclusively to microorganisms that remain viable at the time of consumption and demonstrate documented health-promoting effects. This definition excludes microbial metabolites, non-viable (dead) cells, and fermented products containing undefined microbial consortia. Furthermore, procedures such as fecal microbiota transplantation are not considered probiotics [68].

In order for a microorganism to be classified as a probiotic, it must meet a number of functional and safety criteria. The most important include the ability to survive in gastrointestinal conditions—such as tolerance to low gastric pH and the presence of bile salts—as well as a proven beneficial effect on host health confirmed by both in vitro and in vivo studies [47,67].

Another important aspect is the impact of probiotics on the human body, including modulation of the gut microbiota, stimulation of immune mechanisms, and improvement of nutrient bioavailability. Probiotics have been shown to contribute to reducing the risk of diarrhoea, irritable bowel syndrome, inflammatory bowel diseases, vaginal infections, atopic dermatitis, rheumatoid arthritis, and certain types of cancer [47,69].

Safety is a key factor in qualifying microorganisms as probiotics. According to FAO/WHO and ISAPP guidelines, probiotic strains should have GRAS (Generally Recognized As Safe) status, granted based on laboratory and clinical evidence [67]. This requirement significantly limits the number of microorganisms approved for human use.

Until now, probiotics have primarily included lactic acid bacteria isolated from the human gastrointestinal tract. However, advances in genetic sequencing methods and a deeper understanding of the gut microbiota have led to a reassessment of this approach [68]. Increasingly, microorganisms derived from traditional, spontaneously fermented foods are being recognized as a safe and valuable alternative to classical gut-derived probiotics, provided they meet rigorous safety criteria [67,68].

### 5.2. Next Generation Probiotics (NGPs)

With the advancement of research on the gut microbiota and the development of molecular biology techniques and genetic sequencing, knowledge regarding the diversity of microorganisms influencing human health has significantly expanded. The traditional approach to probiotics, focused primarily on lactic acid bacteria derived from the human gastrointestinal tract, has begun to be perceived as insufficient in light of recent discoveries [67,68].

In response to these limitations, the concept of Next Generation Probiotics (NGPs) has been introduced. This group includes microorganisms that meet the criteria of probiotics but have not yet been widely used in this role [67]. These microorganisms are often isolated from traditional, spontaneously fermented foods of both plant and animal origin [47,67,68].

The primary difference between classical probiotics and NGPs lies in their origin and range of functional properties. Classical probiotics mainly include lactic acid bacteria capable of colonizing the gastrointestinal tract and persisting in the large intestine [67,69]. In contrast, NGPs do not always fulfill this criterion; however, their health-promoting effects may result from high metabolic activity and the production of bioactive compounds [68].

Many authors emphasize that the beneficial health effects attributed to probiotics may be associated not only with the presence of live cells but also with their metabolites or substances released during cell lysis [68]. This observation has led to the emergence of related concepts such as postbiotics, paraprobiotics, and metabiotics, which are often discussed alongside NGPs [68].

Despite the growing number of scientific reports indicating the beneficial effects of NGPs on human health, their practical application in functional foods and medicinal products remains limited. A major barrier is the restrictive regulatory framework established by FAO/WHO, which historically limited probiotic use to microorganisms originating from the human gastrointestinal tract [67].

Additionally, most potential next generation probiotics have not yet been evaluated in a sufficient number of randomized clinical trials, preventing a clear assessment of their safety and efficacy [47,68]. Therefore, the future application of NGPs in food products and nutraceuticals requires further comprehensive research, including studies on host–microorganism interactions and long-term effects [67,68].

### 5.3. Acetic Acid Bacteria as Potential Probiotics

Strains of acetic acid bacteria can be isolated from a variety of sources, including fermented dairy products, vegetables, fruit juices, and traditional fermented foods of both plant and animal origin [47,67,68]. According to current guidelines, microorganisms isolated from fermented foods may be considered potential probiotics, provided they undergo detailed safety and functional evaluation [68]. In this context, AAB are regarded as well-known and safe microorganisms that, in addition to their technological usefulness, also exhibit health-promoting properties [67].

One of the key criteria for classifying microorganisms as probiotics is their ability to survive under gastrointestinal conditions. In vitro studies have shown that selected AAB strains, such as *A. aceti*, *Acetobacter syzygii*, and *Acetobacter indonesiensis*, isolated from fermented dairy products, demonstrate tolerance to low pH and bile salts [47].

Current research indicates that AAB exhibits several characteristics typical of probiotics, including resistance to adverse gastrointestinal conditions, the ability to modulate the gut microbiota, and the production of compounds with documented health benefits [47,57,67].

Despite their limited ability to colonize the gut, AAB meets many functional criteria associated with next generation probiotics. Therefore, there is increasing interest in including selected AAB strains within the NGP category, which may open new perspectives for their application in functional foods and nutraceutical preparations [47,57,67,68].

### 5.4. Probiotic Properties of Acetic Acid Bacteria

One of the fundamental criteria for classifying microorganisms as probiotics is their ability to survive under adverse gastrointestinal conditions, including low pH and the presence of bile salts. Studies have shown that selected strains of acetic acid bacteria, such as *A. aceti*, *A. syzygii*, and *A. indonesiensis*, isolated from fermented dairy products, exhibit high tolerance to these factors [47]. These properties enable AAB to survive gastrointestinal transit and potentially exert effects on the host organism.

Additionally, studies on the strain *A. pasteurianus* BP2201 have confirmed its exceptional resistance to bile acids, supporting its potential probiotic application [57].

Scientific reports indicate that some AAB strains exhibit antimicrobial, anti-inflammatory, and anticancer activities in in vitro studies [47]. These effects may be associated both with the presence of live bacterial cells and with the production of biologically active metabolites [68].

The antimicrobial activity of AAB may contribute to limiting the growth of intestinal pathogens and supporting the body’s natural defense mechanisms [67]. Meanwhile, their anti-inflammatory and anticancer effects suggest potential applications in the prevention of inflammatory and neoplastic diseases of the gastrointestinal tract [47].

Acetic acid bacteria have also demonstrated the ability to reduce oxidative stress, as confirmed in studies on selected AAB strains. The strain *A. pasteurianus* BP2201 showed significant antioxidant activity, contributing to the reduction of oxidative damage in the body [57].

Reduction of oxidative stress is crucial in the prevention of many chronic diseases, including metabolic, neurodegenerative, and neoplastic disorders, highlighting the functional potential of AAB [57,67].

Experimental studies have also shown that selected AAB strains may positively influence lipid metabolism and liver function [57]. The strain *A. pasteurianus* BP2201 was found to reduce blood lipid levels and improve liver damage caused by excessive alcohol consumption.

The ability of AAB to degrade ethanol and modulate oxidative stress may represent an important protective mechanism in alcohol-related diseases, further supporting their therapeutic potential [57].

Despite their limited ability to colonize the large intestine, acetic acid bacteria can modulate the composition of the gut microbiota [57]. This effect may occur through the production of bioactive metabolites, alteration of intestinal environmental conditions, and indirect influence on the growth of commensal bacteria [67,68].

The modulation of gut microbiota by AAB contributes to improved microbial balance in the gastrointestinal tract and may play a role in alleviating metabolic and inflammatory disorders [47,57,68].

### 5.5. Bioactive Metabolites of Acetic Acid Bacteria

#### 5.5.1. Organic Acids and Detoxifying Compounds

Acetic acid bacteria produce a range of compounds with documented health-promoting properties during fermentation, including glucuronic acid (GlcUA), gluconic acid, and D-saccharic acid 1,4-lactone (DSL). DSL, a derivative of D-glucaric acid, exhibits antioxidant and detoxifying properties. It can inhibit glucuronidase activity, thereby supporting glucuronidation processes and enabling more efficient elimination of toxins, tumor promoters, and hepatotoxins from the body [57].

Organic acids produced by AAB also play a role in protecting fatty acids from peroxidation, including polyunsaturated fatty acids, which are essential for proper cell membrane structure and eicosanoid biosynthesis. As a result, AAB metabolites support antioxidant functions and may reduce the risk of chronic diseases such as atherosclerosis and Parkinson’s disease [67].

#### 5.5.2. Hormonal Modulation and Endogenous Metabolism

GlcUA is involved in the elimination of steroid hormones, such as estradiol, through glucuronidation, which may help reduce the toxic effects of hormone excess in the body. Additionally, this metabolite enhances the bioavailability of polyphenols through conjugation, facilitating the transport and absorption of plant-derived bioactive compounds [57].

GlcUA also plays a role in the formation of glycosaminoglycans (GAGs), including hyaluronic acid, chondroitin sulfate, and heparin, which are key components of the extracellular matrix in tissues and organs. These compounds support joint elasticity, connective tissue function, and skin homeostasis [67].

#### 5.5.3. Production of Vitamins and Prebiotics

Some AAB strains are capable of synthesizing vitamin C and levan, a compound with prebiotic properties that supports the growth of beneficial gut microbiota [47]. The production of these bioactive compounds further enhances the functional value of AAB as next generation probiotics.

#### 5.5.4. Therapeutic Potential of Metabolites

AAB metabolites, including organic acids, DSL, GlcUA, vitamin C, and prebiotics, exhibit a broad spectrum of therapeutic effects. These include antioxidant and detoxifying properties, modulation of the gut microbiota, support of liver function, protection of fatty acids from peroxidation, and improved bioavailability of bioactive compounds [47,57,67]. Thus, these metabolites represent an additional mechanism of probiotic action, complementing the effects of live bacterial cells.

### 5.6. Perspectives on the Application of Acetic Acid Bacteria as Probiotics

Selected AAB strains demonstrate high tolerance to low pH and bile salts, enabling their survival in the gastrointestinal tract and indirect modulation of the gut microbiota [47,57]. Additionally, some strains exhibit antimicrobial, anti-inflammatory, and anticancer activities in in vitro studies, as well as the ability to reduce oxidative stress and modulate lipid metabolism and liver function [47,57,67].

These bacteria produce a variety of bioactive metabolites, such as glucuronic acid, DSL, organic acids, vitamin C, and levan, which exhibit detoxifying, antioxidant, and prebiotic properties [47,67]. These metabolites constitute an additional mechanism of probiotic action, complementing the functions of live AAB cells and enhancing their therapeutic potential.

Although AAB does not colonize the large intestine to the same extent as lactic acid bacteria, their functional health effects, microbiota modulation, and production of bioactive compounds support their inclusion in research on modern probiotics. Currently, the main barriers to their full application are the limited number of clinical studies and regulatory constraints related to the safety of new strains [67,68].

Future applications of AAB include the development of functional foods, dietary supplements, and nutraceuticals that may support microbiota balance, metabolic health, liver function, and protection against oxidative stress [57]. Further in vitro and in vivo studies, including randomized clinical trials, are necessary to fully document their safety, efficacy, and therapeutic potential, enabling their broader introduction into the functional food and supplement markets [68].

Despite growing interest in acetic acid bacteria as next generation probiotics, numerous challenges remain regarding their development and application in functional products and dietary supplements [47,68].

Although AAB are well-known microorganisms isolated from traditionally fermented foods, detailed studies on their safety and functional properties are still required [67,68]. Currently, there is insufficient clinical evidence clearly confirming their probiotic effects in humans. Therefore, both in vitro and in vivo studies, including randomized clinical trials, are necessary to evaluate their therapeutic potential, optimal dosages, and mechanisms of action [68].

Another challenge is the comprehensive understanding of the mechanisms of action of AAB and the role of their metabolites, such as glucuronic acid, DSL, organic acids, vitamin C, and levan. While these metabolites have demonstrated antioxidant, detoxifying, and microbiota-modulating properties, further research is needed to assess their bioavailability, interactions with the host, and potential synergistic effects with other gut microorganisms [47,57,67].

As aerobic organisms, AAB do not colonize the large intestine to the same extent as traditional probiotics, such as lactic acid bacteria [67]. Therefore, it is essential to understand how they exert indirect effects on the gut microbiota and host metabolism. Research focusing on indirect mechanisms—such as metabolite production and modulation of the intestinal environment—represents an important direction for future studies [57,68].

Another key challenge is the development of standardized production and quality control methods for AAB strains intended for use in functional foods or probiotic supplements [68]. This includes establishing optimal fermentation conditions, ensuring strain stability in final products, and developing reliable methods for assessing probiotic activity and safety for consumers [68].

Despite these challenges, AAB shows significant potential as components of modern probiotic and functional products. Future research should focus on:

- Isolation of new strains with high probiotic potential;
- Evaluation of their effects in animal and clinical models;
- Investigation of bioactive metabolites and their mechanisms of action;
- Development of safe and effective consumer products [47,57,67,68].

Thanks to these studies, AAB may become an important component of strategies aimed at improving metabolic health, liver function, gut microbiota balance, and protection against oxidative stress, thereby opening insights for their wide application in nutraceuticals and functional foods [57,68].

The potential of AAB in the probiotic and functional food industry is significant, particularly in the context of the growing demand for next generation probiotic products that offer more than traditional lactic acid bacteria [47,68]. The possibility of combining the probiotic properties of live cells with bioactive metabolites creates opportunities for the development of innovative food products and dietary supplements with multifunctional health-promoting effects [47,67,68].

## 6. Applications of Acetic Acid Bacteria in Biotechnology and Bioengineering

Acetic acid bacteria are a unique group of microorganisms with a wide range of applications in biotechnology and bioengineering, resulting from their unique metabolic and biosynthetic capabilities. A characteristic feature of AAB is their ability to convert various substrates, including ethanol, into valuable products such as organic acids, aldehydes, esters, and biopolymers, especially bacterial cellulose (BC) [62,70,71].

Thanks to these properties, AAB are gaining increasing importance in many industries—from the production of ecological preservatives and flavour additives, through the production of pharmaceutical raw materials, to the creation of modern biomaterials for medical and packaging applications [16,62,72]. Thanks to the intensive development of metabolic and synthetic engineering, it is possible to modify these bacteria to increase production efficiency and expand the range of compounds generated, which opens the door to even broader use in the biotechnology industry [28,41].

### 6.1. Bacterial Cellulose (BC) Production

**Figure 7:**
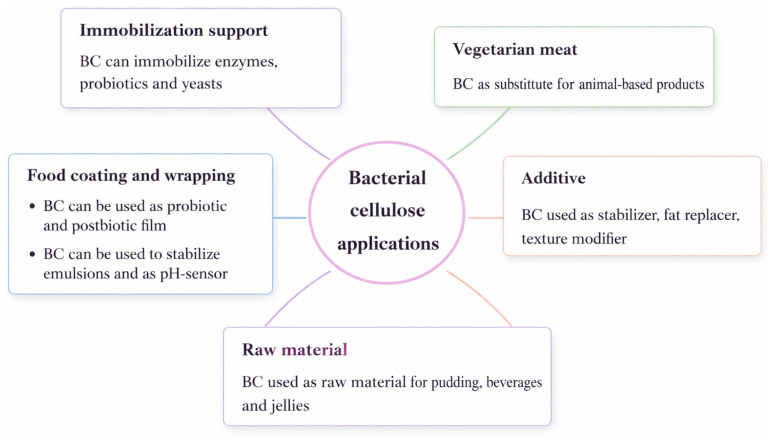
Different applications of bacterial cellulose (BC) in the food industry.

Bacterial cellulose (BC) is a unique biopolymer synthesized by acetic acid bacteria, particularly those of the *Gluconacetobacter* genus (now *Komagataeibacter*). BC is characterized by high purity, mechanical strength, excellent water retention, and a unique fibre nanostructure, making it a material with exceptional physicochemical and biological properties [4,17,18,33,73,74,75]. Figure 7 shows different applications of bacterial cellulose in the food industry.

Bacterial cellulose has several distinctive features that determine its wide application:

- Nanofibrous structure: High porosity and a large specific surface area allow for effective absorption and penetration of substances,
- High purity: BC does not contain lignin, hemicellulose, or other impurities typical of plant cellulose,
- Mechanical strength and flexibility: This allows BC to be used in materials requiring durability and flexibility;
- Biocompatibility and biodegradability: The material is well tolerated by living organisms, opening up a wide range of biomedical applications [4,17,18,33,76].

In medicine, BC is primarily used as a wound dressing material, especially for burns and chronic skin injuries, thanks to its ability to maintain a moist environment and protect against infection [76,77]. BC serves as a basis for creating drug carriers, implants, and artificial tissues due to its low immunogenicity and ability to modify its surface [1,4].

In the food sector, BC is used as a natural thickening agent, stabilizer, and texture-forming ingredient in products such as fermented beverages, desserts, and confectionery [5]. Furthermore, thanks to its barrier properties and biodegradability, BC is an attractive material for the production of edible and eco-friendly protective coatings [5,78].

Bacterial cellulose is gaining increasing importance in the production of biodegradable and functional food packaging, replacing traditional plastics. Thanks to its excellent mechanical strength, transparency, and barrier properties against oxygen and oils, BC is an ideal material for food packaging, which is in line with global pro-environmental trends [5,73,78].

Acetic acid bacteria, as producers of bacterial cellulose, offer a material with unique properties that finds wide application in medicine, the food industry, and ecological packaging solutions. The dynamic development of biotechnology allows for the modification and expansion of BC functionality, increasing its importance in future industrial and medical applications.

### 6.2. Ethanol Bioconversion

Acetic acid bacteria play a key role in the bioconversion of ethanol into a range of valuable chemical compounds that are widely used in the food, pharmaceutical, and chemical industries. This process is based on the specific metabolic pathways of these bacteria, enabling the conversion of alcohol into organic acids, aldehydes, and esters, which exhibit diverse properties and applications [70,79,80].

**Figure 8:**
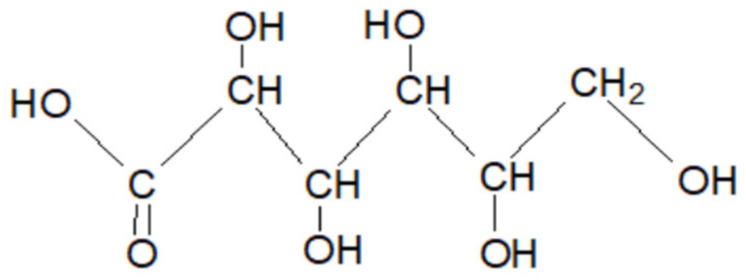
Structural formula of gluconic acid. Created using ChemSketch.

The most well-known and commonly used product of ethanol bioconversion by AAB is acetic acid. Bacteria of the genera *Acetobacter* and *Gluconacetobacter* catalyze the oxidation of ethanol to acetaldehyde and then to acetic acid, which is the main component of table vinegar and a raw material in many industrial processes [4,24]. Furthermore, AAB can also convert ethanol to other organic acids, such as gluconic acid (Figure 8) or hydroxy acids, which are used, among others, as preservatives or chemical precursors [45,71,72].

**Figure 9:**
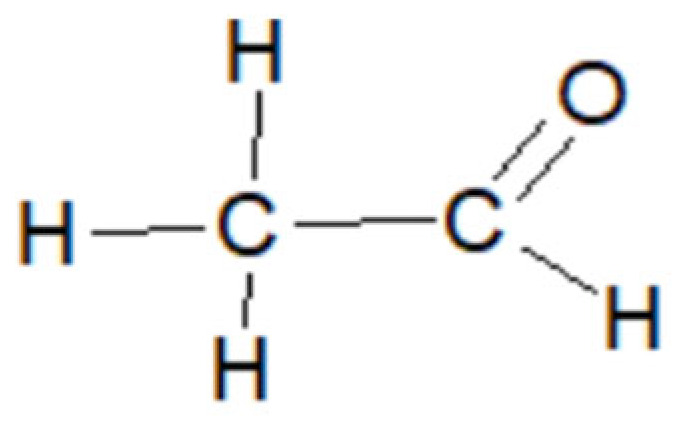
Structural formula of acetaldehyde. Created using ChemSketch software.

AAB are capable of biosynthesizing aldehydes (e.g., acetaldehyde (Figure 9)), which are important intermediates in fermentation and used in the fragrance and pharmaceutical industries [53]. Additionally, through enzymatic condensation of acids and alcohols, these bacteria synthesize esters with characteristic aromas and preservative properties, which are particularly valued in food and cosmetics production [53,81].

Modern metabolic engineering approaches enable increased bioconversion efficiency and a broader range of compounds produced. Genetic modifications of AAB enable the optimization of metabolic pathways, increased production efficiency of acetic acid and other compounds, and the biosynthesis of new bioactive molecules with pharmaceutical potential [41,62].

The bioconversion of ethanol by AAB is the basis for vinegar production, but its significance extends far beyond this traditional application. The produced acids, aldehydes, and esters are used as natural flavour and aroma additives, raw materials for chemical synthesis, and components of pharmaceuticals and cosmetics [62,72,81]. Thanks to the natural nature and high selectivity of the process, the use of AAB in biotechnology is an attractive and ecological alternative to chemical processes [70,71].

### 6.3. Synthesis of Pharmaceutical Compounds

Acetic acid bacteria are valuable microorganisms used as production platforms in pharmaceutical biotechnology, thanks to their ability to biosynthesize a variety of compounds of high biological value. Their use in the production of vitamins, antibiotics, and other bioactive metabolites is particularly important, as they have wide applications in medicine and pharmacy [16,62,82].

**Figure 10:**
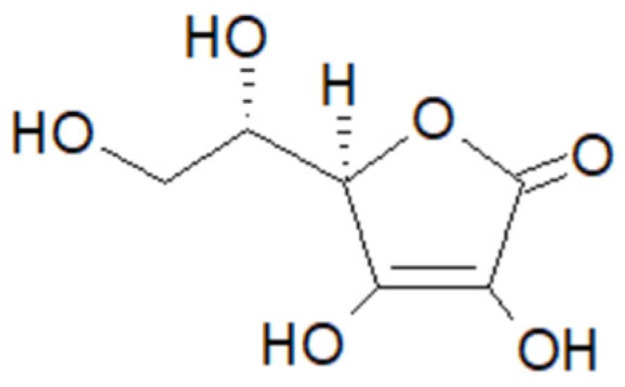
Structural formula of ascorbic acid. Created using ChemSketch.

AAB exhibits the ability to biosynthesize B vitamins and vitamin C precursors, such as ascorbic acid (Figure 10). Thanks to their specific metabolic pathways, these bacteria can be used in industrial processes to produce vitamins of high purity and biological activity, which is crucial for supplementation and drug production [16,56]. Metabolic engineering also enables increased production efficiency of these compounds by optimizing the genes responsible for their biosynthesis [28].

AAB are a source of natural antibiotics and other compounds with antibacterial, antiviral, and antifungal properties. The production of these compounds by bacteria offers a promising alternative to synthetic methods of drug production. These compounds also exhibit immunomodulatory potential, making them attractive candidates for the development of new therapies [56,83]. Despite the challenges associated with production optimization, intensive research on the biotechnological use of AAB is leading to increasingly effective methods for producing bioactive metabolites [28,84].

Advances in synthetic and metabolic engineering are enabling the development of new AAB strains capable of producing highly specialized pharmaceutical compounds. Advanced technologies allow for the manipulation of metabolic pathways, which increases the efficiency of biosynthesis and enables the production of compounds difficult to obtain chemically [28]. Combined with the natural ability of AAB to produce biopolymers such as bacterial cellulose, they provide a universal platform for the production of modern drugs and biomedical materials [4,60,76]. The ability of acetic acid bacteria to produce vitamins, antibiotics, and other biologically active compounds makes them valuable organisms in the pharmaceutical industry. Their biotechnological use enables the development of ecological and economical production processes that could revolutionize the production of drugs and dietary supplements [16,62].

## 7. Applications of Acetic Acid Bacteria in Environmental Protection

Acetic acid bacteria are playing an increasingly important role in modern environmental protection technologies thanks to their unique metabolic properties and ability to process various organic substrates. Their natural activity in the oxidation of alcohols to acetic acid, as well as their ability to form biofilms and function in various environmental conditions, make them valuable tools in biodegradation, bioremediation, and industrial waste disposal processes [85,86,87].

In an era of growing environmental awareness and the need to reduce the negative impact of industrial activities on the environment, AABs are widely used in wastewater treatment, waste processing, and the remediation of areas contaminated with toxic organic substances.

Acetic acid bacteria play a significant role in biotransformation processes and in selected bioremediation systems, particularly in environments rich in ethanol and other simple organic substrates. Thanks to their ability to oxidatively ferment alcohols, AAB are applied in wastewater treatment and industrial waste processing technologies, where they contribute to the conversion of organic compounds into less toxic and more valuable metabolites [88,89].

In biodegradation processes, AAB exhibits metabolic activity primarily associated with the oxidation of alcohols and related compounds, which can support the transformation of organic matter in mixed microbial communities. Their presence in microbial biofilms can enhance the stability and functional performance of such systems, as confirmed in industrial wastewater treatment applications [90,91]. Furthermore, bioaugmentation using AAB may support the degradation of ethanol-rich waste streams, particularly in systems characterized by high concentrations of alcohols [86].

AAB are also involved in the treatment of industrial effluents containing alcohol residues, where microaerobic fermentation enables the conversion of ethanol and other alcohols into acetic acid. This process reduces waste toxicity and generates a metabolite with industrial relevance, thereby supporting sustainable waste management and circular economy approaches [37,88,92].

In wastewater treatment systems with high organic loads, especially those rich in alcohols, AAB contributes to process efficiency through their metabolic activity and tolerance to acidic and alcoholic conditions. Biofilms formed by AAB improve process stability and facilitate synergistic interactions with other microorganisms, enhancing overall degradation performance in engineered systems [5,28].

Acetic acid bacteria are therefore considered important components of biotechnological systems for the treatment of ethanol-rich wastes, contributing to both detoxification and valorization of industrial by-products. The food, alcohol, and biofuel industries generate significant amounts of ethanol-containing waste, and AAB offer promising solutions due to their fermentation and bioconversion capabilities.

AAB are capable of microaerobic oxidation of ethanol and other alcohols into acetic acid, which not only reduces waste toxicity but also yields a valuable industrial product [37,41]. Studies on food waste processing using yeast–AAB co-fermentation have demonstrated efficient conversion of high-alcohol substrates [37]. Additionally, hybrid technologies combining biological and physicochemical processes enable acetic acid recovery from sewage sludge and other industrial residues, as shown in pilot-scale studies [93].

In industrial fermentation systems, ethanol-containing waste can serve as a substrate for microbial bioconversion processes involving AAB, leading to the production of acetic acid and other value-added compounds [28,94]. The ability of AAB to tolerate high concentrations of ethanol, acetic acid, and temperature stress supports their stability in industrial environments with variable operating conditions [95].

An important aspect of these systems is the integration of AAB with other microorganisms in consortia, which can improve substrate utilization and enhance the efficiency of complex waste transformation processes [24,92]. This integrated approach allows for more complete degradation of available organic substrates and contributes to waste minimization.

Overall, fermentation and biotransformation of ethanol-rich industrial residues using acetic acid bacteria represent a sustainable approach for waste management, simultaneously enabling the production of valuable compounds such as acetic acid and supporting environmental and economic objectives.

Acetic acid bacteria play a significant role in biotreatment processes involving high concentrations of alcohols, which are typical for waste streams from food, distillery, and bioethanol industries. Their ability to oxidize ethanol to acetic acid enables the reduction of organic load and toxicity in wastewater systems [41,96].

Studies have shown that AAB form biofilms that contribute to the stability and efficiency of treatment systems such as biofilm reactors and activated sludge processes. These biofilms protect microbial cells from environmental fluctuations and promote interactions with other microorganisms, improving the degradation of organic compounds in mixed cultures [28,80,91].

The tolerance of AAB to ethanol, acetic acid, and elevated temperatures allows their application in treatment systems handling high-strength wastewater, where conventional microorganisms may exhibit reduced activity. These stress-response mechanisms are important for maintaining process stability under industrial conditions [5,95].

The incorporation of AAB into bioaugmentation strategies can enhance the efficiency of wastewater treatment systems, particularly in the removal and transformation of alcohols and related compounds [5,86,97]. Examples of their application include biofilm-based reactors and granular sludge systems, which exploit the natural ability of AAB to form metabolically active aggregates [97,98].

## 8. Applications of Acetic Acid Bacteria in Medicine and Cosmetology

### 8.1. Acetic Acid and Its Antimicrobial Applications

Acetic acid bacteria produce acetic acid, a metabolite with well-documented antimicrobial properties. Acetic acid exhibits broad-spectrum activity against both Gram-positive and Gram-negative bacteria, including clinically relevant pathogens such as *Pseudomonas aeruginosa* and *Staphylococcus aureus* [80]. Its antibacterial effect is primarily associated with acidification of the environment, disruption of membrane integrity, and denaturation of essential cellular proteins, ultimately leading to inhibition of microbial growth and cell death.

Due to these properties, acetic acid has been investigated as a natural antiseptic agent in wound care, dermatology, and infection control. Its effectiveness against antibiotic-resistant strains further increases its potential for clinical applications, particularly in chronic wound management and burn treatment [19,80].

### 8.2. Bacterial Cellulose: Properties and Biomedical Applications

In addition to organic acid production, acetic acid bacteria synthesize bacterial cellulose (BC), a biopolymer with unique structural, mechanical, and biological properties. BC is characterized by high purity, biocompatibility, high water retention capacity, gas permeability, and a nanofibrous structure resembling the extracellular matrix of human tissues [99,100,101].

These properties make BC highly suitable for biomedical applications, particularly in wound healing and tissue regeneration. BC-based dressings create a moist environment that supports epithelialization while acting as a protective barrier against microbial infection [101]. Furthermore, BC can serve as a platform for controlled drug delivery, enabling localized and sustained release of therapeutic agents [99,102].

The functional properties of BC can be further enhanced through modification with bioactive compounds such as chitosan, silver nanoparticles, plant extracts, or antibiotics. Such composites exhibit improved antimicrobial and anti-inflammatory activity and are particularly effective in the treatment of infected or chronic wounds, including those caused by multidrug-resistant organisms such as MRSA [53,103].

In cosmetology, BC is used as a base material for facial masks, hydrogels, serums, and transdermal delivery systems. Its ability to stabilize and gradually release active compounds improves skin hydration, regeneration, and delivery efficiency of cosmetic ingredients [79].

Emerging research focuses on “smart” BC-based materials capable of responding to environmental stimuli such as pH, temperature, or microbial presence, enabling advanced applications in personalized wound care and regenerative medicine [99,104,105].

## 9. Genetic Engineering of Acetic Acid Bacteria

Acetic acid bacteria, particularly strains of the *Komagataeibacter* genus, are an important biotechnological target due to their ability to produce bacterial cellulose (BC) and other valuable metabolites. Genetic engineering of these microorganisms offers extensive opportunities to modify their metabolic pathways, allowing for improved production efficiency, increased tolerance to stress factors, and the introduction of new biochemical functions. Advances in genomic technologies and gene editing tools, such as sRNA-based systems and precise recombination methods, have enabled increasingly precise control of the production of BC and other bioproducts [8,106,107]. However, despite the dynamic development of genetic methods, their application in AAB faces specific challenges stemming from both the unique biology of these bacteria and technical limitations in the transformation and stabilization of introduced genes. Current research focuses on developing regulated gene expression systems that allow for controlled and adaptive adjustment of production levels depending on environmental conditions [34,108]. Furthermore, metabolic engineering of acetic acid bacteria fits into the broader context of synthetic biology and sustainable bioprocesses, enabling the creation of new strain lines with improved production and environmental properties [109,110].

In recent years, genetic engineering of acetic acid bacteria, particularly those from the *Komagataeibacter* genus, has become a key tool for improving bacterial cellulose production and increasing resistance to environmental stressors such as high concentrations of ethanol and acetic acid. Strategic approaches to genetic modification include both classical gene recombination techniques and advanced methods based on the regulation of gene expression and genome editing [8,34,106,108,109]. One innovative approach is the use of RNA interference (sRNA) systems to precisely control the expression of genes involved in cellulose biosynthesis. For example, Florea et al. (2016) demonstrated that implementing synthetic sRNAs allows for the regulation of the activity of BC-synthesizing enzymes, enabling modulation of cellulose production in response to changing environmental conditions [106]. Such regulatory systems increase the production flexibility of BC and provide the basis for the creation of intelligent bioreactors.

Another direction is the development of regulated gene expression systems in *Komagataeibacter.* Research on environmentally or chemically induced promoters (e.g., by IPTG or arabinose) allows for the precise switching on or off of selected genes, which translates into control of cellulose biosynthesis and ethanol metabolism [108]. This approach optimizes production efficiency and minimizes the negative effects of metabolic stress on the cell. Advanced genome editing techniques, including CRISPR-Cas and marker-based selection systems, have been successfully adapted to modify *Komagataeibacter hansenii*, allowing for the precise removal or insertion of DNA fragments. For example, editing cellulose gene clusters allows for optimization of the composition and structure of produced cellulose [8]. Furthermore, introducing genes encoding ethanol-metabolizing enzymes (e.g., adhA and adhB) increases the bacteria’s tolerance to stress associated with high ethanol concentrations and improves production stability [107].

In the context of sustainable BC production, the use of synthetic engineering methods allows for the construction of new metabolic pathways that integrate natural bacterial processes with genetic modules enabling better substrate utilization and increased yield [34,73]. Examples include the introduction of genes enabling the metabolism of a wide range of carbohydrates and improving plasmid stability.

Genetic modification strategies also include approaches based on the optimization of promoter and regulatory element systems, which reduces cell burden and improves the stability of recombinant structures. Work on increasing gene stability in the bacterial genome or on plasmids, as well as eliminating natural restriction systems that hinder transformation, are other important aspects in the development of effective genetic engineering methods for AAB [106,111].

In recent years, significant attention has also been paid to co-culture techniques, where genetically modified strains cooperate with other microorganisms, thereby circumventing the challenges associated with genetic transformation and increasing the efficiency of BC production [112].

Strategies for genetically modifying acetic acid bacteria combine traditional recombinant methods with modern tools of synthetic biology and genome editing. Despite numerous technical challenges, advances in gene expression regulation, structural stability, and metabolic engineering have enabled significant increases in bacterial cellulose production efficiency and opened up new opportunities for the development of innovative applications of this biopolymer in medicine, the food, and packaging industries [8,107,108,109,111,112].

Genetic engineering of acetic acid bacteria, especially strains of the *Komagataeibacter* genus, faces a number of significant limitations and technological challenges that hinder the full utilization of these microorganisms’ potential in biotechnology. One of the main problems is the low efficiency of genetic transformation, resulting, among other things, from the bacteria’s natural defence mechanisms, such as restriction-modification systems, which eliminate foreign DNA, significantly limiting the possibility of introducing desired genes or plasmids [106,113]. Furthermore, the stability of the introduced genetic constructs in AAB cells is often insufficient—the presence of plasmids is associated with the risk of mutation, sequence loss, or metabolic burden, which can lead to decreased production of bacterial cellulose or other metabolites [111]. Another challenge is the specific structure and physiology of *Komagataeibacter* cells, which exhibit resistance to many standard transformation methods, such as electroporation and conjugation, hindering the efficient introduction of genetic material [49]. The need to optimize transformation protocols for each strain significantly increases the time and cost of engineering [106,108]. Furthermore, the expression of foreign genes in AAB requires precise control of expression regulation, and available regulatory systems are still under development, limiting the ability to dynamically adapt the production of biopolymers or metabolites to changing environmental conditions [34,106,108].

Another technological limitation is the so-called plasmid burden, which results from the intensive expression of genes on plasmids and can negatively impact bacterial growth and production efficiency. Excessive expression of proteins or enzymes can lead to cellular stress and metabolic disruptions, requiring careful selection of promoters and expression systems [112]. Genetic manipulations that interfere with key metabolic pathways can also cause unforeseen side effects, limiting the stability and reproducibility of the resulting strains [107]. Furthermore, due to limited genetic tools and the lack of comprehensive synthetic engineering systems dedicated to AAB, alternative approaches, such as co-cultures, are often used. These approaches bypass direct genetic modification but have their own limitations related to process control and scalability [100,112].

## 10. Safety of Acetic Acid Bacteria

The safety of acetic acid bacteria and their metabolic products is a key aspect in the context of the widespread use of these microorganisms in the food, pharmaceutical, and biotechnology industries. Among the most important products manufactured by AAB is bacterial cellulose, which, thanks to its unique physicochemical properties and biocompatibility, has been used as a biodegradable packaging material, a food ingredient, and a medical biomaterial [32,72,114,115,116].

Multidimensional toxicological studies, including the assessment of acute toxicity, cytotoxicity, genotoxicity, and antigenotoxic properties of exopolysaccharides and bacterial cellulose, confirm their safety and lack of harmful effects on living organisms [31,39,115,117]. Furthermore, advanced BC production and functionalization technologies enable the production of materials of high purity and quality control, which is essential to meeting regulatory requirements in the European Union, the United States, and other regions of the world [32,116,118].

The safety of acetic acid bacteria and their metabolites is a key aspect in the context of their wide applications, particularly in medicine, cosmetology, and the food industry. Numerous studies have shown that AAB, and in particular the bacterial cellulose they produce, are characterized by very low toxicity and lack of pathogenic properties, confirming their high biocompatibility and safety of use [114,115,117]. Toxicological studies of bacterial cellulose obtained from sugarcane molasses have demonstrated a lack of toxic effects at acute toxicity, cytotoxicity, and genotoxicity levels, confirming its potential for safe use in contact with skin and tissues [117]. Furthermore, toxicological assessment of bacterial cellulose conducted on both cell models (endothelial cells) and animal organisms confirms its biocompatibility and lack of negative impact on living tissues [115]. Bacterial cellulose is characterized by a unique nanofibrous structure that promotes cell proliferation and minimizes the risk of inducing immune reactions or inflammation, which is important for its use in medical dressings and cosmetic products [32,73,114]. The high purity and absence of bacterial contamination or endotoxins in AAB preparations further confirm their safety and low risk of pathogenicity. It is also worth emphasizing that biotechnological processes for bacterial cellulose production utilize methods that allow for obtaining a material with a high degree of sterility and purity, which is crucial in eliminating potential toxicological hazards. Furthermore, the biodegradable properties of bacterial cellulose enable its safe degradation in the biological environment, reducing the risk of accumulation and potential toxic effects [32,116,118].

The available scientific data clearly indicate that acetic acid bacteria and their metabolites, particularly bacterial cellulose, exhibit a very low toxicity profile and lack of pathogenicity. Therefore, they can be safely used in various fields, including dermatological, medical, and food applications, as confirmed by numerous toxicological and biocompatibility studies [31,114,115,117].

The safety of acetic acid bacteria and their metabolites in various applications, including the food and medical industries, is strictly regulated by relevant legal standards and food and drug safety standards. One of the key concepts in this regard is the GRAS (Generally Recognized As Safe) status, awarded by the U.S. Food and Drug Administration (FDA), which signifies that the substance is generally recognized as safe for use based on broad scientific consensus and available toxicological studies [31,117].

Acetic acid fermentation products, including bacterial cellulose (BC) produced by AAB, have in many cases been granted GRAS status, confirming their safety for food and pharmaceutical applications. In particular, BC is increasingly used as a biodegradable packaging material and a carrier for active substances in food products, which requires meeting stringent sanitary and toxicological standards [32,118]. Toxicological studies indicate the absence of adverse effects and the lack of pathogenicity of these materials, which is crucial for obtaining a positive regulatory assessment [115,117].

In the European Union, the safety of products containing AAB metabolites is subject to Regulation (EU) No. 2015/2283 on novel foods and EFSA (European Food Safety Authority) regulations. Bacterial cellulose is classified as a low-risk ingredient, facilitating its introduction to the market as a food ingredient or functional additive [115,118]. Additionally, production processes and purification methods must meet GMP (Good Manufacturing Practice) requirements, ensuring consistent product quality and safety [116]. For medical applications such as wound dressings or drug carriers, AAB and their metabolites must comply with ISO standards and FDA requirements for Class II and III medical devices. Numerous studies on AAB functionalization confirm its biocompatibility and lack of toxicity, which is necessary for obtaining certificates of conformity and approval for clinical use [72,114,115]. In summary, acetic acid bacteria and their fermentation products, particularly bacterial cellulose, are considered safe in accordance with applicable international standards and legal regulations (GRAS status, EU regulations, FDA). Their compliance with regulations is confirmed by a wide range of toxicological studies and positive opinions from regulatory authorities, allowing for their widespread use in the food, medical, and cosmetics industries [31,32,39,117,118].

In addition to their generally recognized safety profile, recent studies have highlighted a potential concern regarding the presence of antibiotic resistance determinants in acetic acid bacteria isolated from fermented foods. Genomic and phenotypic analyses of vinegar-associated strains have revealed resistance to certain antibiotics, although the clinical relevance of these traits and the likelihood of horizontal gene transfer to pathogenic microorganisms remain uncertain and require further investigation. This aspect should be considered in the context of the potential use of AAB as next-generation probiotics and food-grade microbial cultures [84].

## 11. Future and Directions of Research on Acetic Acid Bacteria

Acetic acid bacteria are an important element of modern biotechnology due to their ability to produce bacterial cellulose (BC) and high-quality vinegar. Faced with growing industrial needs and requirements for sustainable production, AAB research focuses on optimizing production processes, implementing modern technologies, and expanding the range of applications for these microorganisms. Scientific developments in these areas allow for the potential of AAB to be utilized in new, innovative applications, which constitutes a promising research direction in the coming years [116,119,120].

One of the most important areas of research is the optimization of bacterial culture conditions to maximize the yield of AAB and vinegar. Optimal carbon sources, culture media, pH, temperature, and other fermentation parameters are crucial for process efficiency [120,121,122]. For example, studies have shown that a variety of substrates, such as agave juice or agricultural waste, can significantly increase BC production by *K. hansenii* strains [123]. Simultaneously, work on optimizing vinegar production focuses on increasing the strains’ tolerance to high concentrations of ethanol and acetic acid and improving bioconversion efficiency [8].

In the era of digitalization and the development of information technology, artificial intelligence is finding increasingly widespread applications in biotechnology. In BC production processes, AI is used to model and optimize fermentation conditions, predict yields, and control parameters in real time [124]. Modern bioreactors, equipped with advanced monitoring and automation systems, enable precise control of environmental conditions, significantly improving production stability and repeatability [116,125]. Integrating these technologies with synthetic biology enables the creation of hybrid production systems that are more efficient and environmentally friendly [126]. Potential for using acetic acid bacteria in biofactories to produce high-value-added compounds.

Besides the production of BC and vinegar, BC has the potential to be used as biofactories for the production of various bioactive compounds, such as enzymes, polymers, and pharmaceuticals. Metabolic and synthetic engineering allows for the modification of the metabolic pathways of these bacteria to obtain new or improved high-value-added products [120]. This approach could contribute to the development of sustainable biotechnology based on renewable raw materials and environmentally friendly processes [119,127].

## 12. Summary

Acetic acid bacteria (AAB) represent a metabolically diverse group of microorganisms with growing importance in food biotechnology and sustainable food production. Their oxidative metabolism, driven by membrane-bound dehydrogenases and tightly regulated by oxygen availability, supports the formation of key metabolites such as acetic acid, gluconic acids, and bacterial cellulose. These compounds contribute not only to food preservation and safety but also to sensory quality, texture development, and the creation of clean-label fermented products. As consumer demand for minimally processed and naturally preserved foods increases, AAB-based bioprocesses offer valuable opportunities for innovation in the food sector.

Beyond their technological relevance, emerging evidence highlights the potential of selected AAB strains to exert health-promoting effects. Their ability to produce organic acids, exopolysaccharides, and other bioactive metabolites positions them as promising candidates for functional foods, postbiotic formulations, and microbiome-targeted dietary strategies. AAB may support gut microbial balance, enhance intestinal barrier integrity, and modulate host immune responses, although these mechanisms require further elucidation. Their role in non-traditional fermentations—such as kombucha, water kefir, and novel plant-based beverages—further expands their relevance in the development of next-generation functional foods.

Recent advances in metabolic engineering, systems biology, and synthetic biology have significantly broadened the biotechnological potential of AAB, enabling improved yields, enhanced stress tolerance, and more efficient oxidative bioprocessing. However, several limitations remain, including strain-dependent genetic instability, limited availability of standardized genetic tools, and challenges in controlling oxygen transfer and metabolic fluxes during large-scale fermentation.

Future research should prioritize:

- The development of robust and universal genetic engineering platforms for AAB;
- Optimization of bioreactor design and oxygen management strategies tailored to oxidative fermentations;
- Comprehensive characterization of probiotic and postbiotic properties of AAB using in vitro, in vivo, and clinical approaches;
- Integration of multi-omics, metabolic modeling, and machine learning for rational strain design;
- Exploration of AAB in novel food matrices, plant-based substrates, and low-waste circular bioprocesses.

Collectively, these efforts will support the broader application of AAB as microbial cell factories and functional microorganisms in sustainable food production, health-promoting foods, and microbiome-oriented nutritional interventions.

Overall, AAB constitutes promising microbial cell factories for the sustainable production of organic acids, biopolymers, and other high-value compounds, while also emerging as potential functional microorganisms with applications in nutrition, health promotion, and gut microbiome modulation.

## Figures and Tables

**Table 1:** Differential characteristics of the genera *Acetobacter*, *Gluconacetobacter*, *Gluconobacter*, and *Komagataeibacter* [29].

Characteristic	*Acetobacter*	*Gluconobacter*	*Gluconacetobacter*	*Komagataeibacter*
Motility and flagellation	peritrichous or non-motile	polar or non-motile	peritrichous or non-motile	no
Oxidation of ethanol to acetic acid	+	+	+	+
Oxidation of acetic acid to CO_2_ and H_2_O	+	-	+	+
Oxidation of lactate to CO_2_ and H_2_O	+	-	+	+
Growth on 0.35% acetic-acid-containing medium	+	+	+	+
Growth in the presence of 30% d-glucose	-	+ or -	+ or -	n.d.
Production of cellulose	-	-	+ or -	+ or -
Acid production from:				
Glycerol	+ or -	+	+	n.d.
d-Mannitol	+ or -	+	+ or -	-
Raffinose	-	-	-	n.d.
Production from D-glucose of:				
2-keto-D-gluconic acid	+ or -	+	+ or -	+ or -
5-keto-D-gluconic acid	+ or -	+ or -	+ or -	+ or -
2,5-keto-D-gluconic acid	+ or -	+ or -	+ or -	-
Ubiquinone type	Q9	Q10	Q10	Q10

**Table 2:** Selected species of acetic acid bacteria and their sources in food products [60].

Product	AAB Species
Bacterial cellulose	*Novacetimonas hansenii* (formerly *Komagataeibacter hansenii*), *Komagataeibacter nataicola*, *Komagataeibacter rhaeticus*, *Komagataeibacter swingsii*, *Komagataeibacter maltaceti*, *Komagataeibacter xylinus*
Cereal vinegar	*Komagataeibacter europaeus*, *Komagataeibacter kakiaceti*, *Komagataeibacter medellinensis*
Cheese whey vinegar	*Acetobacter aceti*, *Acetobacter pasteurianus*
Cocoa	*Acetobacter pasteurianus*, *Acetobacter syzygii*, *Acetobacter tropicalis*, *Acetobacter ghanensis*, *Acetobacter indonesiensis*, *Acetobacter okinawensis*, *Komagataeibacter hansenii*, *Gluconobacter oxydans*, *Gluconobacter frateurii*, *Gluconacetobacter diazotrophicus*, *Granulibacter bethesdensis*
Fruit vinegar	*Acetobacter pasteurianus*, *Acetobacter aceti*, *Acetobacter estunensis*, *Acetobacter pomorum*, *Acetobacter syzygii*, *Komagataeibacter europaeus*, *Komagataeibacter hansenii*, *Komagataeibacter kakiaceti*, *Komagataeibacter oboediens*, *Komagataeibacter maltaceti*, *Komagataeibacter melomenusus*, *Komagataeibacter pomaceti*, *Komagataeibacter rhaeticus*, *Komagataeibacter saccharivorans*, *Gluconacetobacter entanii*, *Novacetimonas maltaceti*, *Komagataeibacter nataicola*, *Komagataeibacter intermedius*, *Komagataeibacter xylinus*, *Gluconobacter oxydans*
Gluconic acid, dihydroxyacetone, vitamin C precursors, miglitol	*Gluconobacter oxydans*
Kombucha	*Acetobacter papayae*, *Acetobacter indonesiensis*, *Acetobacter lovaniensis*, *Acetobacter okinawensis*, *Acetobacter peroxydans*, *Acetobacter syzygii*, *Acetobacter tropicalis*, *Komagataeibacter takamatsuzukensis*, *Komagataeibacter oboediens*, *Komagataeibacter europaeus*, *Komagataeibacter saccharivorans*, *Komagataeibacter intermedius*, *Komagataeibacter xylinus*, *Komagataeibacter rhaeticus*, *Komagataeibacter hansenii*, *Gluconacetobacter liquefaciens*, *Gluconacetobacter entanii*, *Gluconobacter cerinus*, *Gluconobacter oxydans*, *Tanticharoenia sakaeratensis*
Lambic	*Acetobacter orientalis*, *Gluconobacter cerevisiae*, *Gluconobacter wancherniae*, *Acetobacter pasteurianus*, *Acetobacter aceti*, *Acetobacter lovaniensis*, *Acetobacter lambici*, *Acetobacter pomorum*
*Levan*	*Gluconobacter albidus*, *Gluconobacter cerinus*, *Gluconobacter oxydans*, *Gluconobacter frateurii*, *Kozakia baliensis*, *Neoasaia chiangmaiensis*, *Tanticharoenia sakaeratensis*, *Komagataeibacter hansenii*, *Komagataeibacter xylinus*, *Acetobacter pasteurianus*, *Gluconacetobacter diazotrophicus*
Water kefir	*Acetobacter indonesiensis*, *Acetobacter fabarum*, *Acetobacter orientalis*, *Acetobacter tropicalis*, *Acetobacter okinawensis*, *Acetobacter lovaniensis*, *Komagataeibacter intermedius*, *Komagataeibacter hansenii*, *Komagataeibacter saccharivorans*, *Gluconobacter cerinus*, *Gluconobacter japonicus*, *Gluconacetobacter liquefaciens*

## Data Availability

No new data were created or analyzed in this study. Data sharing is not applicable to this article.
